# Novel Formulations of Sourdough Bread Based on Supplements Containing Chokeberry Juice Fermented by Potentially Probiotic *L. paracasei* SP5

**DOI:** 10.3390/foods13244031

**Published:** 2024-12-13

**Authors:** Ioanna Mantzourani, Maria Daoutidou, Antonia Terpou, Stavros Plessas

**Affiliations:** 1 Laboratory of Food Processing, Department of Agricultural Development, Democritus University of Thrace, 68200 Orestiada, Greece; imantzou@agro.duth.gr (I.M.);; 2 Department of Agricultural Development, Agri-Food, and Natural Resources Management, School of Agricultural Development, Nutrition & Sustainability, National and Kapodistrian University of Athens, Evripos Campus, 34400 Evia, Greece; aterpou@agro.uoa.gr; 3 Laboratory of Microbiology, Biotechnology and Hygiene, Department of Agricultural Development, Democritus University of Thrace, 68200 Orestiada, Greece

**Keywords:** sourdough bread, nutritional value, chokeberry juice, supplement, phytic acid

## Abstract

The current study focused on sourdough breads produced with various supplements consisting of freeze-dried black chokeberry juice, (i) unfermented and (ii) fermented by *Lactiplantibacillus paracasei* SP5, aiming to enhance their functionality and nutritional value. Specifically, the impact of these supplements on the quality of sourdough breads was evaluated in terms of their nutritional features, antimicrobial capacity, and sensorial characteristics. Sourdough breads produced with freeze-dried fermented chokeberry juice exhibited elevated concentrations of lactic acid (2.82–2.99 g/kg) and acetic acid (0.93–0.99 g/kg), which significantly prolonged their resistance to mould growth and rope contamination, maintaining freshness for over 13 days. These samples also demonstrated higher antioxidant activity, with DPPH values exceeding 4 μmol TE/g and ABTS values surpassing 218 mg TE/100 g, along with a total phenolic content ranging from 85.9 to 96.3 mg GAE/100 g. Additionally, these samples showed a greater reduction in phytate, an antinutrient, compared to all other samples, including the control. The sensory evaluation conducted with consumer panels indicated that sourdough breads prepared with freeze-dried fermented chokeberry juice achieved the highest ratings in terms of taste and appearance among all tested samples. The findings are highly promising and suggest the potential for commercializing the developed supplements in the production of additive-free sourdough bread with enhanced nutritional value.

## 1. Introduction

Over the past decade, innovations in the food industry have significantly advanced, focusing primarily on enhancing nutritional value, extending shelf-life, improving sensory attributes, and developing additive-free, safe food products [1,2,3,4]. The baking industry is no exception to this trend. Among the various techniques employed, sourdough technology stands out as a widely utilized method, offering well-documented benefits, such as enhanced nutritional content, prolonged shelf life, superior quality attributes, and potential health advantages [5,6,7]. In addition to the application of sourdough technology, other biotechnological methods and strategies have been recently proposed and developed in the bread production industry. Specifically, the addition of supplements (mainly in dried powdered form) of high nutritional value seems to be quite an attractive method for ameliorating the nutritional value of bread and delivering possible health benefits [8,9,10]. The combination of sourdough technology with the appropriate supplemental addition is even more effective. In this vein, there are some examples in the recent bibliography which reveal the advantages of this new trend [6,11,12,13].

Several supplements, including powdered cinnamon, chokeberry pomace, pomegranate peel, grape extracts, and others, have been explored for their application in bread production [14,15,16]. The main outcome of these research efforts was the improvements in technological properties and nutritional value of produced breads. However, in some instances, other critical objectives, such as achieving accepted sensorial features or further improving nutritional profiles, were not fully met, indicating the need for continued research in this area [8,17]. 

Recent research has explored the development of innovative sourdough breads incorporating freeze-dried supplements, such as pomegranate juice and its fermented counterpart produced with *Lactiplantibacillus plantarum* subsp. *plantarum* ATCC 14917. These approaches have shown promise in enhancing both the functionality and sensory attributes of sourdough products. Furthermore, these breads demonstrated increased resistance to microbial spoilage compared to the control, an outcome attributed to higher acidity, elevated organic acid concentrations, and the potential antimicrobial effects of pomegranate-derived phenolics [9]. The concept of utilizing freeze-dried fermented fruit juice processed by lactic acid bacteria (LAB) appears to be a viable and innovative alternative that merits further investigation. Building on this, recent work with black chokeberry juice fermented by the potentially probiotic strain *Lactiplantibacillus paracasei* SP5 resulted in a functional product characterized by enhanced total phenolic content (TPC) and antioxidant activity (AC) [18]. Black chokeberry, also known as *Aronia melanocarpa*, is considered as a highly nutritive fruit, with high levels of functional polyphenols [19]. Even though chokeberry has been applied as a powdered supplement in the bread production industry [17], it has not been applied in the form of juice nor in sourdough technology, only in yeast-leavened bread [20,21,22,23,24].

The objective of the current study was to investigate, for the first time, the potential use of (i) unfermented chokeberry juice and (ii) chokeberry juice fermented by *Lactiplantibacillus paracasei* SP5 as innovative, nutrient-rich supplements in the production of sourdough bread. The supplements were incorporated into the sourdough bread formulation in a powdered form using lyophilization to ensure stability and ease of application. The resulting breads were subjected to a thorough evaluation, encompassing a wide range of parameters, including physicochemical properties (such as texture and moisture content), microbiological stability (resistance to contamination and spoilage), nutritional composition (protein, phenolic content, and antioxidant activity), and sensory attributes (taste, aroma, and texture). Furthermore, the study investigates the economic viability and practicality of scaling-up the production and integration of these supplements in commercial sourdough bread manufacturing, considering factors such as cost-efficiency, supply chain availability, and consumer acceptance.

## 2. Materials and Methods

### 2.1. Substrates, Microorganism, and Cultivation Media

The recently isolated LAB strain *L. paracasei* SP5, obtained from kefir grains, was employed to ferment black chokeberry juice, which was then utilized in the production of sourdough bread. The strain was cultivated at 37 °C for 48 h in MRS broth, and the biomass was harvested via centrifugation at 5000 rpm for 10 min at 25 °C (Sigma 3K12, Βioblock Scientific, Sigma Larborzentrifugen GmbH, Osterode, Germany) under sterile conditions.

The preparation of black chokeberry juice followed the previously described protocol [18]. In brief, black chokeberries (*Aronia melanocarpa*) were sourced from a local organic farm in Orestiada, Greece. The juice was extracted and sterilized; deionized water was added to achieve an initial sugar concentration of 40 g/L, with no additional sugars incorporated.

The fermentation process of the chokeberry juice adhered to the previously established method [18]. The juice was first heated at 80 °C for 10 min. and then cooled to approximately 37 °C. Next, 1 g of freeze-dried biomass containing viable *L. paracasei* SP5 cells (approx. 8.2 log cfu/g) was added per 100 mL of the fermentation medium. The pH of the substrate was then adjusted to 4.0 ± 0.1 by the addition of sterile 0.1 M NaOH.

For bread production, commercial wheat flour (ELBISCO CO S.A., Halkida plant, Glyfa, 341 00, Greece) was employed, characterized by the following composition (% *w/w*): 12.0% moisture, 72.0% carbohydrates, 11.0% protein, 1.5% fat, and 2.2% fibre. Baker’s yeast (S.I. Lesaffre, France) was provided in pressed block form, containing 70% moisture.

### 2.2. Freeze-Drying

The freeze-drying process of both chokeberry juice and *L. paracasei* SP5-fermented juice was carried out by initially freezing the samples at −44 °C with a cooling rate of 5 °C/min. The drying step was conducted for 48 h at a pressure range of 5–15 mbar and a temperature of −45 °C using a Freeze-Drying System (FreeZone 4.5, Labconco, Kansas City, MO, USA) [1]. The resulting freeze-dried chokeberry juice (UFCJ) and fermented chokeberry juice (FCJ) were subsequently incorporated as functional supplements in the production of sourdough bread.

### 2.3. Sourdough Bread Making

**Figure 1:**
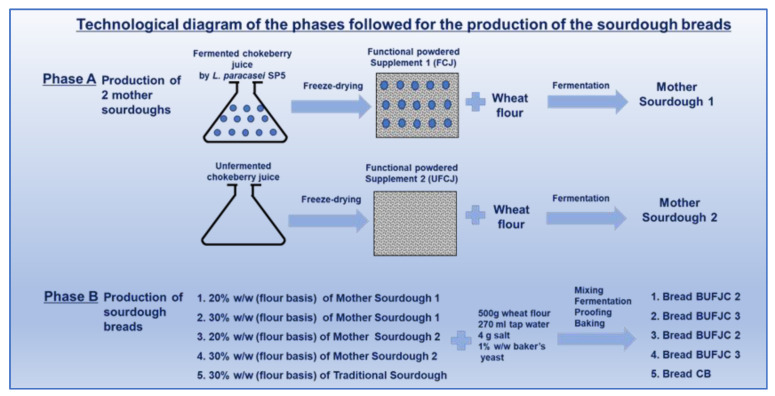
Technological diagram of the phases followed for the production of sourdough breads.

The complete procedure for sourdough bread production is illustrated in Figure 1. Initially, two mother sponges were prepared by mixing 300 g of wheat flour with 160 mL of tap water for 15 min. The mixtures were prepared as follows: (i) with 1% *w/w* freeze-dried fermented chokeberry juice (FCJ, based on flour weight), and (ii) with 1% *w/w* freeze-dried unfermented chokeberry juice (UFCJ, based on flour weight). Subsequently, four types of sourdough breads were produced using mother sponges containing 20% and 30% *w/w* (based on flour weight) of the aforementioned sponges. Specifically, four bread variants were formulated as follows: (i) 20% UFCJ (BUFJC2), (ii) 30% UFCJ (BUFJC3), (iii) 20% FCJ (BFJC2), and (iv) 30% FCJ (BFJC3). Each bread dough included the specified content of the corresponding sourdough, 500 g of wheat flour, 270 mL of tap water, and 4 g of salt. The doughs were placed into 1.5 L baking pans. Additionally, 1% *w/w* of pressed baker’s yeast (based on flour weight) was incorporated into all doughs. The doughs underwent fermentation at 30 °C for 2 h, followed by proofing at 40 °C for approximately 60 min, and baking at 230 °C for approximately 40 min. A control bread (CB) was also prepared using traditional sourdough sourced from a local bakery, which contained 30% traditional sourdough (on flour basis). The formulation and procedure for the control bread followed the same steps as described for the sourdough breads containing freeze-dried chokeberry juice with *L. paracasei* SP5. All trials were carried out in triplicate.

### 2.4. Analytical Methods

#### 2.4.1. Microbial Cell Counts and Monitoring of Bread Spoilage

The concentration of viable cells (colony-forming units, cfu/g) in the freeze-dried *L. paracasei* SP5 and fermented chokeberry juice powders was assessed by homogenizing 1 g of the sample in 9 mL of a phosphate buffer (KH_2_PO_4_, 0.25 M solution diluted at 1.25 mL per litre of distilled water, pH 6.5) [9]. Briefly, the resulting suspension was serially diluted and plated. Lactic acid bacteria (LAB) were identified on MRS agar (Fluka, Buchs, Switzerland) and incubated at 37 °C for 48 h, while yeasts were identified on malt agar (Fluka, Buchs, Switzerland) after a 2-day incubation at 30 °C. Spoilage was assessed macroscopically according to previously established methods [25].

#### 2.4.2. Organic Acids

The concentrations of organic acids (lactic, acetic, formic, propionic, n-valeric, and caproic) in the sourdough breads were analyzed using HPLC, following the method described previously [9].

#### 2.4.3. pH and Total Titratable Acidity (TTA)

The pH and total titratable acidity (TTA) levels of the sourdough bread samples were measured using the method previously outlined, which involved a systematic analysis to assess the acidity characteristics and overall sourness of the bread matrix [9].

#### 2.4.4. Specific Loaf Volume

The specific loaf volume (mL/g) of the sourdough bread samples was determined using the rapeseed displacement method, a technique that involves measuring the volume displaced by rapeseeds when the bread sample is submerged, thereby providing an accurate assessment of the bread’s porosity and overall structure [9].

#### 2.4.5. Total Phenolic Content (TPC)

After baking was completed, the breads were allowed to cool at room temperature for 3 h to ensure stabilization. Subsequently, the breads were sliced, and bread crumb samples were subjected to freeze-drying for 48 h to preserve their biochemical composition. Then, 1 g of the freeze-dried sample was suspended in 20 mL of phosphate-buffered saline (PBS, pH 7.4) and incubated for 1 h at 37 °C under continuous shaking to facilitate the extraction of soluble compounds. The resulting extracts were separated by transfusion, and the remaining residues were re-extracted with an additional 20 mL of PBS. The extracts obtained from these steps were then combined and stored at -20 °C until further analysis for total phenolic content (TPC) and antioxidant activity (AC).

The TPC (mg GAE/100 g of dried sample) was quantified using the Folin–Ciocalteu reagent method, as previously described in the established literature [9].

#### 2.4.6. Antioxidant Capacity (AC)

The antioxidant capacity (AC) of the bread extracts was evaluated using two well-established methods. First, the ABTS [2,2′-azino-bis (3-ethylbenzothiazoline-6-sulfonic acid)] assay was employed to measure the Trolox Equivalent Antioxidant Capacity (TEAC), which provides a quantitative assessment of the sample’s ability to scavenge free radicals. Second, the DPPH (2,2-diphenyl-1-picrylhydrazyl) radical scavenging activity assay was performed to determine the capacity of the extracts to neutralize free radicals. Both assays were conducted following previously described protocols, ensuring accurate and reliable measurements of the antioxidant properties present in the bread extracts [9].

#### 2.4.7. Phytic Acid

Phytic acid, also known as myo-inositol-1,2,3,4,5,6-hexakisphosphate, was quantified by measuring the phosphorus released as a result of enzymatic activity. This analysis was conducted using the Megazyme K-PHYT test kit, which is a reliable and widely accepted method for assessing phytic acid content. The procedure followed the manufacturer’s instructions (Megazyme, Bray, Ireland) to ensure accurate and reproducible measurements. The test effectively determines the concentration of phytic acid by breaking it down enzymatically and releasing inorganic phosphorus, which is then quantified as an indicator of the phytic acid content in the sample.

#### 2.4.8. Sensory Evaluation

A blind sensory evaluation of the organoleptic quality of all sourdough breads was conducted immediately after their production. A panel of 20 randomly selected, untrained testers assessed the breads based on a nine-point hedonic scale, ranging from 1 (dislike extremely) to 9 (like extremely). This evaluation focused on key sensory attributes, including taste, aroma, texture, and appearance. The methodology followed established procedures as described in previous studies, ensuring a standardized assessment to determine consumer preference and overall acceptance of the bread samples [9].

#### 2.4.9. Statistical Analysis

Statistical analysis was conducted using Analysis of Variance (ANOVA), followed by Duncan’s post hoc multiple range test, with a significance level set at 5%. The data analysis was carried out using the SPSS Statistics 20.0 software (IBM Corp., Armonk, NY, USA), maintaining an alpha level of 5% throughout the evaluation.

## 3. Results and Discussion

In the context of the present study, five distinct sourdough bread formulations were developed to evaluate the impact of incorporating different types of chokeberry juice supplements on bread quality. These formulations included a control bread containing a traditional commercial sourdough (CB), as well as sourdough breads containing unfermented chokeberry juice, at concentrations of 20% and 30% *w/w* (referred to as BUFJC2 and BUFJC3, respectively). Additionally, sourdough breads containing freeze-dried, fermented chokeberry juice processed with the probiotic strain *L. paracasei* SP5 were prepared at 20% and 30% *w/w* (designated as BFJC2 and BFJC3, respectively).

The study aimed to comprehensively evaluate the effects of these supplements on multiple bread characteristics. Specifically, various physicochemical properties, including loaf volume, crumb structure, and texture, were assessed. The phytic acid content, an important antinutrient affecting mineral bioavailability, was quantified to determine nutritional implications. Additionally, sensory attributes, such as taste, aroma, texture, and visual appeal, were evaluated to assess consumer acceptance and preference. Finally, the preservation performance of each bread formulation was monitored over time to evaluate their shelf life and resistance to spoilage.

The experimental findings are presented in detail and analyzed to provide insights into the potential benefits and challenges of incorporating unfermented and fermented chokeberry juice supplements into sourdough bread production. The results are discussed in terms of technological, nutritional, and sensory impacts, as well as their implications for both bread quality enhancement and commercial viability.

### 3.1. Viable Cell Counts in the Sourdoughs

The populations of lactic acid bacteria (LAB) and yeasts were quantified in all the produced sourdough samples (Table 1). While the viable yeast counts were comparable across all sourdoughs and showed no significant statistical differences, notable variations were observed in the population of viable LAB counts. In particular, the addition of chokeberry juice, fermented or not, significantly increases the viability of LAB in the respective sourdoughs, compared to the commercial sourdough. Sourdough prepared with fermented chokeberry juice contained 10.3 log cfu/mL LAB, followed by sourdough containing unfermented chokeberry juice (8.7 log cfu/mL) and commercial sourdough (8.2 log cfu/mL). Even though the explanation of high levels of LAB in the sourdough containing fermented chokeberry juice can be attributed to the presence of LAB in the juice, the higher viability of LAB in sourdough containing unfermented chokeberry juice compared to commercial sourdough implied possible prebiotic properties of chokeberry juice [26,27,28]. This can be verified by the recent international bibliography, since the phenolics that contained in high amounts in chokeberry juice were included in the recent definition of prebiotics, enhancing in that way the growth of probiotics [29]. However, the prebiotic action of the juice in the current study is unclear due to the limited sample size.

### 3.2. Quality Properties of Sourdough Bread

The physicochemical characteristics of the produced sourdough breads were assessed (Table 2). Notably, the specific loaf volume remained relatively consistent across all samples, with no statistically significant differences observed, ranging between 2.54 and 2.61 mL/g. A similar outcome was sought in many other cases since an equal content of yeast (responsible for bread rising) was added in the process of all bread production without any significant difference in the initial sourdoughs. The levels of SLV (2.5–3 mL/g) are rational, as many other studies have revealed, taking into account approximately the same operational conditions [1].

Notably, substantial statistical differences (*p* < 0.05) were observed in the acidity and pH values across all the sourdough bread samples produced in this study. Among the samples, the BFJC3 variant demonstrated the highest acidity, reaching 9.92 mL NaOH 0.1 M, along with the lowest pH value of 4.35. This sample was followed by the BFJC2 sample, the two BUFJC samples, and, lastly, the control bread sample (CB). A similar pattern was evident in the organic acid profiles, where the BFJC3 sample exhibited higher concentrations of the primary organic acids, lactic acids, and acetic acids, in comparison to the other samples. Specifically, lactic acid was present at 2.99 g/kg bread, while acetic acid was detected at 0.99 g/kg bread. Furthermore, minor organic acids, such as formic, n-valeric, and caproic acids, were also found in elevated concentrations of 0.15, 0.13, and 0.08 g/kg bread, respectively. It is obvious that the addition of fermented chokeberry juice significantly enhanced the acidity and organic acid levels of the produced breads. This observation is consistent with findings from previous studies, where a powdered supplement composed of freeze-dried fermented pomegranate juice, produced with *L. plantarum* ATCC 14907, was incorporated into sourdough bread production [9]. The underlying explanation for this outcome can be attributed to the elevated levels of lactic acid bacteria (LAB) observed in the initial sourdough cultures prior to bread production. Furthermore, the matrix of chokeberry fruit contains prebiotic compounds, primarily polyphenols, which may positively influence the proliferation of LAB, including probiotic strains such as *L. plantarum* ATCC 14907 and *L. paracasei* SP5 [30,31]. This observation can be more declarative in the case of BUFJC samples. In this case, LABs are absent in the microbiological composition of this supplement. Similarly, the lactic acid bacteria (LAB) naturally present in the flour used for sourdough production are stimulated by the components of chokeberry juice, resulting in higher growth levels compared to those observed in the commercial sourdough (Table 1).

### 3.3. Appearance of Mould and Rope Spoilage

**Figure 2:**
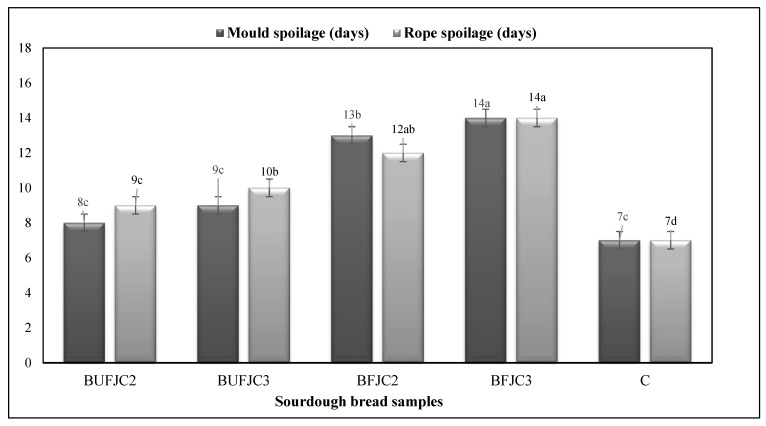
Development of mould and rope spoilage in sourdough bread samples. Different superscript letters in a column indicate statistically significant differences (ANOVA, Duncan’s multiple range test, *p* < 0.05).

The results concerning the development of mould and rope spoilage in all sourdough bread samples are shown in Figure 2. The BFJC3 sample demonstrated the highest resistance to both rope and mould spoilage, with spoilage manifestations occurring around the 14th day. In comparison, the BFJC3 sample showed spoilage on days 12 and 13, respectively. Furthermore, the incorporation of freeze-dried unfermented chokeberry juice resulted in a delayed onset of mould and rope spoilage, which was statistically significantly different from the control sample (CB). The higher content of main and minor organic acids in BFJC3 rationally led to advanced resistance of the respective breads, as other reports have verified [32,33]. Even though lactic and acetic acids are responsible for the high antimicrobial and antifungal properties in sourdough bread, other minor organic acids such as formic, valeric and caproic acid may act in a co-operative mode and further contribute to the delay of the microbial spoilage of breads [34].

### 3.4. Total Phenolic Content and Antioxidant Activity Analysis

Notable findings were observed in the analysis of total phenolic content (TPC) and antioxidant activity (AC) across all sourdough bread samples (Table 3). Specifically, all samples demonstrated statistically significant differences (*p* < 0.05). The BFJC3 samples posed the highest AC (239.2 mg TE/100 g in terms of ABTS and 4.9 μmol TE/g in terms of DPPH) and TPC (96.3 mg GAE/100 g) values, followed by BFJC2, BUFJC2, BUFJC3, and finally, CB. The explication of this interesting result is due to the chemical composition of chokeberry juice and the presence of *L. paracasei* SP5. The use of a component of high phenolic content leads to the ameriolation of the viability of LAB [12,17,20,35,36]. In addition, the degradation of initial phenolic compounds to reduced phenolic compounds leads to elevated AC and TPC levels [12,17,20,35,36]. Incorporating supplements with high phenolic and antioxidant contents into breadmaking typically results in an increase in total phenolic content (TPC) and antioxidant activity (AC) [12,17,20,35,36].

In the present study, the freeze-dried supplement is based on chokeberry juice, which exhibits high phenolic content. The addition of powdered pomace or whole chokeberry fruit can ameliorate the antioxidant activity and total phenolic content in white bread as has been reported in a few other studies [20,21,37]. The main difference in the present study is that the bread was produced with the sourdough technology and that the chokeberry was added in the form of juice. This phenomenon may account for some significant differences in the sensory attributes, which will be discussed in detail later. Furthermore, recent studies have reported that lactic acid fermentation of chokeberry juice leads to an increase in both total phenolic content (TPC) and antioxidant activity (AC) [18]. This effect can be further justified in the case of the sourdough bread produced by the addition of unfermented aronia juice. Indeed, TPC and AC loads were determined in higher levels in BUFJC2 and BUFJC3 samples compared to CB, implicating the direct effect of chokeberry juice. However, more studies are needed in this field in order to determine the kind and the levels of the produced phenolic compounds.

In addition, the action of *L. paracasei* SP5 seems to further influence the TPC and AC levels of bread samples, as can be observed through the comparison of BUFJC and BFJC samples (Table 3). Although unfermented chokeberry juice contains antioxidant compounds, the conversion of these compounds into simpler forms with enhanced antioxidant properties is likely less pronounced compared to the transformation observed in fermented chokeberry juice produced with *L. paracasei* SP5. *Lactobacillus* strains are known to possess substantial capabilities for phenolic biotransformation [38,39]. Additionally, phenolic acids have the potential to act as free radical scavengers, exhibiting significant antioxidant activity [40]. *L. paracasei* SP5 was proven to have this ability, since its application in lactic acid fermentation of chokeberry juice led to a product with improved TPC contents and antioxidant activity [18].

### 3.5. Concentration of Phytic Acid in Samples

The inherent presence of phytic acid detected in cereal products appears to affect the absorption of minerals like Cu, Zn, Co, and Mn by forming complexes with these elements [41]. Likewise, the reduction in this compound is considered as an important target in order to overcome the low absorptions and increase the nutritional value of cereal products, such as bread. Enzymatic actions, through lactic acid fermentation and decreases in the pH value, are very important tools in order to moderate the action of phytic acid [42].

**Figure 3:**
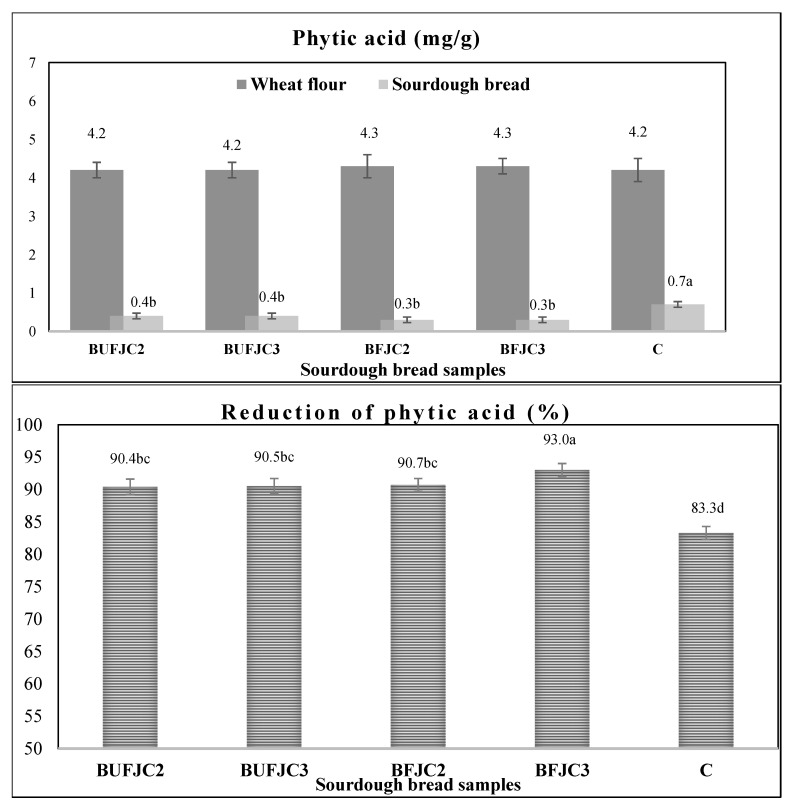
Phytic acid content (increased) and phytic acid reduction (decreased) in dough samples before and after the baking process. Different superscript letters in a column indicate statistically significant differences (ANOVA, Duncan’s multiple range test, *p* < 0.05).

Sourdough breads produced through the proposed supplements exhibited underlined reduced contents of phytic acid, probably due to better action of intrinsic cereal phytases (Figure 3). In particular, all sourdough bread samples containing fermented or unfermented supplements displayed a higher reduction in phytic acid compared to CB. Among them, BFJC3 achieved the highest reduction (93%), followed by BFJC2, BUFJC2, and BUFJC3 (approximately 90%). Last but not least, possible extracellular phytase activity by *L. paracasei* SP5 may also be a reason for the higher reduction in phytic acid, as it has been reported lately [43].

### 3.6. Organoleptic Assessment Focusing on Consumer Preference Evaluation

The outcomes of the sensory evaluation, which reflect consumer preferences for the sourdough breads produced in this study, are presented in Table 4. A detailed comparison highlighted significant differences in both the appearance and taste characteristics of the various bread samples. In particular, the BFsJC2 and BFJC3 sourdough breads were rated highest for taste attributes (*p* < 0.05), showcasing superior flavour profiles that were well-received by the participants. On the other hand, the BFJC3 sample stood out with the highest score for appearance characteristics (*p* < 0.05), indicating a visually more appealing product compared to the other bread samples.

It is worth noting that despite these positive sensory outcomes, previous research has shown that incorporating chokeberry in bread production, particularly in the form of freeze-dried pomace or whole fruit, can sometimes result in undesirable organoleptic changes. Such studies have reported negative impacts on taste and texture, highlighting the challenges of maintaining favourable sensory properties when using chokeberry as an ingredient [20,37]. Nonetheless, in this study, the use of freeze-dried fermented chokeberry juice with *L. paracasei* SP5 appears to have enhanced the sensory characteristics, indicating a more favourable integration of this supplement into sourdough bread formulations. However, this effect on bread characteristics, as observed in previous studies, was not evident in the current investigation.

The observed differences in sensory features may be attributed to two main factors: (i) the application of the sourdough technology in the present study, which, in general, ameliorates the sensorial features of the produced bread [13,44,45], in contrast to the direct yeasted method, and (ii) the use of chokeberry in the form of juice rather than its whole or by-product forms, such as peel or pomace.

## 4. Conclusions

The incorporation of the novel supplement, consisting of fermented chokeberry juice containing the potentially probiotic strain L. paracasei SP5, into sourdough bread production resulted in enhanced nutritional characteristics of the final product. Specifically, there was an observed increase in antioxidant capacity (AC) and total phenolic content (TPC), alongside a reduction in phytic acid concentration. Additionally, the final bread exhibited superior resistance to rope and mould spoilage, persisting up to the 14th day, which can be attributed to the elevated concentrations of both major and minor organic acids. Preliminary sensory evaluations further demonstrated that the final product achieved the highest scores for taste and appearance attributes, highlighting the positive impact of the supplement on both organoleptic and functional bread characteristics. The proposed supplement did not affect the physicochemical features of the product (e.g., loaf volume).

The findings presented here offer promising insights for extending this research into other areas, such as clinical studies, as the nutritional intervention involving sourdough bread enriched with plant-based ingredients—like chokeberry juice, which is abundant in natural antioxidants (phenolic compounds, polyphenols, and flavonoids)—may serve as a valuable tool in managing various health conditions, including type 2 diabetes [46]. Furthermore, the integration of the proposed freeze-dried supplements into the bread industry, particularly for gluten-free formulations, holds significant potential. These supplements can be incorporated in diverse formulations while maintaining their functional properties over extended periods, thereby ensuring long-term stability and efficacy.

## Figures and Tables

**Table 1:** Quantification of Yeast and Lactic Acid Bacteria (LAB) populations in the produced sourdough samples.

Sourdough	Log cfu/g	
	LAB	Yeasts
UFJC	8.7 ± 0.2 ^b^	7.5 ± 0.1 ^c^
FJC	10.3 ± 0.1 ^a^	7.9 ± 0.1 ^a^
C	8.2 ± 0.1 ^c^	7.8 ± 0.2 ^a^

LAB: lactic acid bacteria; cfu: colony-forming units. Distinct superscript letters within a column indicate statistically significant differences (*p* < 0.05).

**Table 2:** Physicochemical properties of sourdough breads.

Bread Sample	pH	TTA (mL 0.1 M NaOH/10 g)	SLV (mL/g)	Content of Organic Acids (g/kg Bread)				
				Lactic	Acetic	Formic	n-Valeric	Caproic
BUFJC2	4.79 ± 0.04 ^a^	6.41 ± 0.05 ^d^	2.56 ± 0.07 ^a^	2.43 ± 0.07 ^c^	0.75 ± 0.01 ^d^	tr	tr	tr
BUFJC3	4.64 ± 0.03 ^b^	7.85 ± 0.05 ^c^	2.57 ± 0.04 ^a^	2.55 ± 0.07 ^c^	0.81 ± 0.02 ^c^	0.08 ± 0.01 ^b^	0.08 ± 0.01 ^b^	0.04 ± 0.01 ^b^
BFJC2	4.50 ± 0.04 ^c^	8.49 ± 0.05 ^b^	2.54 ± 0.07 ^a^	2.82 ± 0.07 ^b^	0.93 ± 0.01 ^b^	0.09 ± 0.01 ^b^	0.08 ± 0.01 ^b^	0.04 ± 0.01 ^b^
BFJC3	4.35 ± 0.04 ^d^	9.92 ± 0.09 ^a^	2.59 ± 0.07 ^a^	2.99 ± 0.04 ^a^	0.99 ± 0.01 ^a^	0.15 ± 0.01 ^a^	0.13 ± 0.01 ^a^	0.08 ± 0.01 ^a^
CB	4.70 ± 0.05 ^ab^	6.23 ± 0.08 ^e^	2.61 ± 0.04 ^a^	2.11 ± 0.05 ^d^	0.70 ± 0.02 ^e^	tr	tr	tr

TTA: total titratable acidity; SLV: specific loaf volume; tr: traces (<0.01 g/kg). Different superscript letters within a column denote statistically significant differences (ANOVA, Duncan’s multiple range test, *p* < 0.05).

**Table 3:** Total Phenolic Content (TPC) and Antioxidant Activity (AC) in sourdough breads expressed on a dry weight basis.

Sourdough Bread	TPC	AC	
		ABTS	DPPH
	(mg GAE/100 g)	(mg TE/100 g)	(μmol TE/g)
BUFJC2	69.0 ± 2.6 ^d^	189.7 ± 3.4 ^d^	3.4 ± 0.1 ^d^
BUFJC3	75.4 ± 2.9 ^c^	199.1 ± 4.1 ^c^	3.8 ± 0.2 ^c^
BFJC2	85.9 ± 4.0 ^b^	218.4 ± 3.8 ^b^	4.3 ± 0.1 ^b^
BFJC3	96.3 ± 3.7 ^a^	239.2 ± 4.9 ^a^	4.9 ± 0.2 ^a^
CB	55.0 ± 3.1 ^e^	183.4 ± 3.9 ^d^	3.2 ± 0.1 ^c^

Different superscript letters in a column indicate statistically significant differences (ANOVA, Duncan’s multiple range test, *p* < 0.05).

**Table 4:** Sensory evaluation results (consumer preference) of sourdough breads.

Sourdough Bread Sample	Aroma	Taste	Appearance
BUFJC2	8.6 ± 0.2 ^a^	8.8 ± 0.1 ^b^	8.4 ± 0.2 ^b^
BUFJC3	8.6 ± 0.2 ^a^	8.8 ± 0.1 ^b^	8.4 ± 0.1 ^b^
BFJC2	8.7 ± 0.1 ^a^	9.4 ± 0.2 ^a^	8.7 ± 0.2 ^b^
BFJC3	8.9 ± 0.1 ^a^	9.5 ± 0.2 ^a^	9.0 ± 0.1 ^a^
CB	8.7 ± 0.1 ^a^	8.8 ± 0.1 ^b^	8.5 ± 0.1 ^b^

Different superscript letters in a column indicate statistically significant differences (ANOVA, Duncan’s multiple range test, *p* < 0.05).

## Data Availability

The original contributions presented in the study are included in the article, further inquiries can be directed to the corresponding authors.
